# Editorial: Scalable Network Generation & Analysis

**DOI:** 10.3389/fdata.2022.984256

**Published:** 2022-07-25

**Authors:** Philippe J. Giabbanelli, Samarth Swarup, Renaud Lambiotte, Giuseppe Mangioni

**Affiliations:** ^1^Department of Computer Science & Software Engineering, Miami University, Oxford, OH, United States; ^2^Biocomplexity Institute, University of Virginia, Charlottesville, VA, United States; ^3^Mathematical Institute, University of Oxford, Oxford, United Kingdom; ^4^Department of Electric Engineering, Electronics and Informatics, University of Catania, Catania, Italy

**Keywords:** big data analytics, graph mining, network analysis, network simulation, scalable algorithms

Network models have long been employed to test theories or provide synthetic datasets for simulations. With the *growing availability of hardware* such as Graphical Processing Units (GPUs), there is a paradigm shift in generating instances of network models *via* algorithms that efficiently leverage distributed computing resources. A familiar approach to characterize the instances of a network model (e.g., in terms of clustering or average distance) has been to provide a closed form analysis, hence using graph theory to demonstrate the behavior of a model. With the explosion of *machine learning* research, the classical matter of characterizing instances of a model has now been recast into a prediction problem that involves training over a massive number of network instances. Analysts have been accustomed to examining networks by looking for the “usual suspects”, such as nodes with high betweenness or closeness centrality. But today's toolbox is overflowing with hundreds of node centrality algorithms, hence a chief issue for analysts is to *select metrics* that will provide an informative answer on their data within a reasonable time. *Scalability* is at the heart of these multiple transformations witnessed in network science research, from the generation or analysis of massive networks to the production of a massive number of networks for machine learning. In this special issue, fifteen authors contribute to guiding the network science research community by proposing practical solutions that address a shared concern for scalability.

Innovative practices for large-scale network generations are proposed by Alam and Perumalla. The authors produced a generator that can realize any desired degree distribution, thus making it a particularly flexible tool for common tasks such as generating equivalent random networks (e.g., to test the presence of a property in an empirical network) or creating a synthetic population. Most importantly, the generator can achieve rates of over 50 billion edges per second through its high utilization of a single modern GPU. By creating the first GPU-based algorithm to generate networks with a given degree distribution, this work is also an invitation for the network science community to explore numerous potential extensions, such as using multiple GPUs (on the same machine and/or *via* an interconnection network) to realize even larger networks in a timely manner.

The creation of a network generator is often accompanied by a characterization of its instances based on the generator's parameters. This task can be arduous as the apparent simplicity of a generator's rules can lead to highly intricate instances once these rules are applied repeatedly, particularly in the presence of stochasticity or bifurcations. The cornerstone of the novel approach offered by Murase et al. consists of creating a massive number of instances from the generators and to use them as training data for a machine learning model that is then able to predict an instance's characteristic from the parameter values. This creative re-purposing of deep neural networks is applied to a new generator, which combines mechanisms that tend to be found in separate generators such as triadic closure, homophilic interactions, and link termination.

In order to adequately cover the enormous potential of machine learning in network science research, the work of Murase et al. is complemented by two studies. Adiga et al. also trained a machine learning algorithm to make predictions based on model parameters, but the prediction here focuses on a dynamical property (spread of invasive species). The study thus illustrates the usefulness of machine learning in investigating the classic matter of structure and function in networks. Another facet is represented by the work of Kejriwal, who focuses on link prediction. In this situation, machine learning serves to predict the existence of a network element rather than properties (whether static or dynamic) of the entire network. This work also reminds readers of this special issue that machine learning in network science can involve diverse types of data. Indeed, the study demonstrates the value of Natural Language Processing (NLP) techniques as they outperform network-theoretic models in predictions on empirical data.

The ability to analyze empirical network data at scale is a key concern for Freund and Giabbanelli, whose study examines the runtime of 18 contemporary node centrality measures based on the properties of a network (e.g., small-world, scale-free, random). The study relies on a carefully crafted process to generate networks of different properties that have a comparable density, hence isolating the effect of a property onto the runtime of a centrality algorithm. This emphasizes the importance of network generation, which has been a recurring theme across studies in this special issue. The study also noted the potential to use GPUs as a means of accelerating the computation of node centralities, hence going back to the first paper on GPU-acceleration. This example, among others, reminds readers that the articles of this special issue can certainly be read independently, but future innovations are likely to be achieved when considering them together. Indeed, this special issue is rich from the diversity of the articles, which are united by their focus on scalability but approach it through different methods. We thus hope that this combination of shared aims and diverse skillsets will provide an inspiration to the research community and facilitate a fruitful exchange of ideas.

## Author contributions

PG has written the editorial. All authors approved the submitted version.

## Conflict of interest

The authors declare that the research was conducted in the absence of any commercial or financial relationships that could be construed as a potential conflict of interest.

## Publisher's note

All claims expressed in this article are solely those of the authors and do not necessarily represent those of their affiliated organizations, or those of the publisher, the editors and the reviewers. Any product that may be evaluated in this article, or claim that may be made by its manufacturer, is not guaranteed or endorsed by the publisher.

